# Mechanism-informed machine learning for interpretable drug release prediction and virtual formulation intervention in polymeric long-acting injectables

**DOI:** 10.3389/fmed.2026.1958605

**Published:** 2026-09-28

**Authors:** Mohammed Ghazwani, Yahia Alghazwani, Umme Hani

**Affiliations:** 1 Department of Pharmaceutics, College of Pharmacy, King Khalid University, Abha, Saudi Arabia; 2 Department of Pharmacology, College of Pharmacy, King Khalid University, Abha, Saudi Arabia

**Keywords:** drug release prediction, explainable artificial intelligence, knowledge-guided graph, machine learning, polymeric long-acting injectables

## Abstract

The correct time-course forecasting of the release of a drug contained in a drug-polymer matrix is difficult because drug dissolution is a complex process involving all formulation parameters, which are interdependent and have nonlinear effects. Although the predictive accuracy of machine learning (ML) models is high, classical methods generally cannot differentiate between predictive features that are only correlated and actually useful ones that are relevant in a formulation design. This work presents a framework for mechanism-informed ML that brings together pharmaceutical knowledge, a knowledge-driven graph, explainability of artificial intelligence (AI), intervention-aligned effect estimation, and computer-assisted formulation experiments. The XGBoost model, which used data on physicochemical properties, composition, and time, showed good results (R^2^ = 0.9774, RMSE = 0.0491; MAE = 0.0339). Feature analysis showed that correlation and SHAP importance agree strongly (r = 0.761), while both correlation and SHAP importance for effects under intervention are very poor (r = 0.132 and 0.021), indicating that predictive and actionable variables differ substantially. This new platform facilitates evaluating formulation changes thoroughly and can help decide which experiments to prioritize, resulting in LAI development of polymeric material being transparent and based on mechanism.

## 1 Introduction

Polymeric LAIs have established themselves as an essential drug delivery platform by maintaining continuous therapeutic levels, decreasing the need for frequent dosing, enhancing patient compliance, and minimizing the impact of unwanted side effects. Different classes of polymeric materials are in play, and it is the biodegradable ones that are commonly used due to their controlled disintegration rate and high level of biocompatibility. Examples include PLGA (1, 2). The main challenge is how different features of the polymer, properties of the drug, formulation, and experimental conditions interplay to decide how the drug will be delivered over time. It is a combination of several factors such as polymer molecular size, the ratio between lactide and glycolide, drug amount, molecular size, hydrophobicity, and release time that determine the behavior of polymer breakdown, drug permeabilization, and erosion, making it quite difficult for any prediction method to accurately model or extrapolate (3). Polymer formulation development used to primarily depend on repeated tests and trial-and-error refinement to get the desired result, which is quite tedious and costly and results in prolonged product development time (4). The introduction of computational methods as support tools in this domain has opened up a new area where these methods not only help to explain the relation between formulation components and performance features but also reduce the time needed for developing a new formulation (5).

ML, in particular, can be a powerful aid in handling complex formulation problems where the relationship between formulation variables is highly nonlinear and hard to predict (6). However, these models typically only learn and extrapolate statistical patterns from training data, which does not allow them to recognize true cause-and-effect relationships but rather only highlight spurious correlations. It can therefore be safely assumed that even though such highly accurate prediction models might exist, they are not the right ones that can give a sound mechanistic explanation or even be used for making formulation decisions on rational grounds (7, 8). Recently, mechanism-aware artificial intelligence has demonstrated that incorporating domain knowledge into learning algorithms can lead to better model robustness, interpretability, and scientific consistency. Apart from just discovering correlations, if one combines pharmaceutical knowledge with causal reasoning, then it becomes possible to figure out which variables truly are the reason for the effect of a drug and also predict, for example, how the change of a single factor will impact the whole formulation (9). The fact is that this sort of skillset is extremely useful in pharmaceutical formulation optimization, where it is as essential to uncover the causal relationship of formulation parameters as it is to build a model with very good predictive power (10). In this context, new opportunities are emerging for integrating causal inference and ML in pharmaceutical research.

Advances in ML have been key in improving the estimation of drug release from LAI at the polymeric level. The results of the first studies showed that ML algorithms are very suitable for capturing formulation-property-performance links, thus allowing the workload of developing a formulation and the time it takes to bring a new product to the market to be significantly shortened (11). By using a large dataset of 1, 537 LAI formulations, the hybrid ML models presented by Wang et al. showed excellent performance with an R^2^ of about 0.86; the SHAP analysis revealed that assay conditions could, indeed, impact drug release to the same extent as formulation variables, thus highlighting the necessity of standardized experimental protocols (12). In their research, Robles and Samad suggested a framework for explainable ML that can predict drug release profiles without the specification of time as a separate input variable. The polymer characteristics, namely molecular weight, the encapsulation efficiency value (EE%), and the physicochemical drug properties, turned out to be some of the most important factors that influence release behavior (13). Drawing from both classification and regression, one such two-stage system successfully demonstrated the power of the XGBoost technique in the case of predicting the dissolution of PLGA microspheres through the provision of a SHAP analysis, which, in turn, revealed the mechanistic features (14). Besides being predictive tools, ML models have also been a vehicle for incorporating scientific knowledge directly into the learning algorithm through integration of scientific equations. By merging mass-transfer equations with AI models, controlled-release predictions have been made more accurate and the model itself more interpretable (15).

A physics-enhanced approach to multi-objective ML, which embeds mechanistic aspects into prediction, has improved accuracy for drug metabolism prediction (16). At the same time, general discussions point to the necessity of transparent AI in formulating medicines as well as getting approval from regulatory agencies (17). In addition, recent methodological advances have focused on causal inference as a way of going beyond merely predicting based on correlation. Exhaustive reviews of causal ML have confirmed its usefulness in the scientific decision-making context through estimating individual treatment effects (2); on the other hand, the thorough evaluation of causal discovery algorithms has been of great assistance in choosing methodologies like PC, NOTEARS, and DirectLiNGAM for datasets with numerical values (18). In a parallel manner, generative AI has emerged as a powerful tool for computational formulation optimization and virtual product design (19). However, despite these advances, an important gap remains in ML-based drug-release prediction for polymeric LAIs. Existing studies have primarily focused on predictive accuracy and feature importance, using ML and explainable AI to identify variables associated with drug-release behavior. These approaches, however, do not necessarily determine whether a highly predictive variable is also an effective target for formulation intervention. Predictive importance may reflect correlation, proxy relationships, or mediation rather than the expected effect of deliberately changing a formulation variable. Meanwhile, causal discovery, treatment-effect estimation, and virtual formulation optimization have generally been investigated as separate approaches rather than within an integrated framework for polymeric LAI drug release. Thus, the key unresolved question is whether the variables that are most useful for predicting drug release are also the variables that can most effectively be manipulated to alter release behavior.

To address this gap, this study develops a mechanism-informed ML framework that integrates pharmaceutical knowledge, causal structure discovery, predictive ML, explainable AI, intervention-effect estimation, and counterfactual formulation analysis. The framework combines a knowledge-informed DAG with PC, NOTEARS, and DirectLiNGAM for causal structure analysis, XGBoost for drug-release prediction, and ATE/CATE-based analyses for evaluating formulation interventions. Counterfactual analysis is then used to virtually evaluate formulation changes. In this way, the proposed framework distinguishes predictive importance from intervention relevance, providing an interpretable basis for formulation analysis and virtual experimental prioritization. This work has several major scientific contributions:

- Building a system that couples the pharmaceutical knowledge domain with causal inference for LAI drug release modeling.
- Development of a DAG that contains mechanism information that was improved through the application of data-driven causal discovery techniques, e.g., PCNOTEARS and DirectLiNGAM.
- Causal treatment of a given situation was estimated via different methods such as ATE, CATE, and individual treatment effects to quantify the effects of changing the composition of a formulation.
- Incorporation of causal thinking into the use of ML models led to a new type of drug release modeling that would support scientific discoveries and understanding through causality instead of being purely data-driven.
- Counterfactual formulation analysis was introduced, which allows virtual optimization and also simulation of changes in the formulation.

Beyond predictive accuracy, the framework opens a new direction for pharmaceutical AI: explainable AI systems that rationally formulate, design experiments, reduce required trials, and speed up the development of long-acting drugs.

## 2 Dataset

### 2.1 Data source and construction

The source data for this framework were collected originally by Bannigan et al. for their ML investigation on polymeric LAIs (20). The dataset is a combination of experimental data from the authors' research group as well as formulations reported in the literature, searched for at the Web of Science site mainly using these search terms: “polymeric microparticle” and “drug delivery.” Most articles only showed drug release profiles as graphs; because of that, the researchers retrieved fractional release values by extracting them from the figures (using GetData Graph Digitizer software) instead of relying on the raw data. Ultimately, the study is based on 181 drug release profiles, with 3,783 separate fractional release measurements across 43 different drug-polymer pairs. The LAIs are mainly microparticle or cylindrical implant formulations made of commercial polymers, mostly PLGA, with additional polylactic acid (PLA) and polycaprolactone (PCL), spanning various molecular weights; when possible, also the compositions (i.e., lactide-to-glycolide ratios).

### 2.2 Feature Set

Initially, 17 descriptors were chosen according to pharmaceutical subject matter expertise to characterize each formulation instance; but in the end, after redundancy analysis (Section 2.4), only 15 remained. The descriptors fall into the four categories that are part of the four descriptors of the causality framework: *Polymer properties;* polymer molecular weight (Polymer_MW), lactide-to-glycolide ratio (LA/GA, zero for non-PLGA systems), and molar cross-linking ratio (CL_Ratio, zero for non-crosslinked systems). One drug-polymer grouping variable is also preserved by the original authors. This is analogous to the DP_Group descriptor. But they used it more for group-wise splitting than as direct model inputs. *Drug properties:* molecular weight (Drug_MW), topological polar surface area (Drug_TPSA), number of heteroatoms (Drug_NHA), melting temperature (Drug_Tm), acid dissociation constant (Drug_pKa), calculated octanol-water partition coefficient (Drug_LogP). Except for Tm and pKa, source publications were used for the others, while descriptors from SMILES strings were computed using RDKit. *Formulation variables:* initial drug-to-polymer ratio (Initial D/M ratio), drug loading capacity (DLC), surface area-to-volume ratio (SA-V, introduced by the original authors to unify spherical and cylindrical geometries in one model), and surfactant concentration in the release medium (SE, zero when absent). *Experimental and prior-release variables:* elapsed time (Time) and fractional release at three early time points, 6 h (T = 0.25), 12 h (T = 0.5), and 24 h (T = 1.0). When early time-point data were lacking, the authors filled in missing values with the best-fitting polynomial over the 0–2-day period.

### 2.3 Dataset characterization and feature preprocessing

Before model development, the dataset was subjected to several preprocessing steps to improve data quality and ensure compatibility with the ML algorithms. Duplicate records were removed to prevent repeated observations from disproportionately influencing model learning. Missing numerical values were imputed using the median of the corresponding feature because the median is less sensitive to extreme observations than the mean and is therefore appropriate for formulation-related variables that may exhibit skewed distributions. Categorical variables were converted using one-hot encoding to represent formulation and experimental categories without imposing an artificial ordinal relationship. Finally, numerical variables were standardized to place features on comparable scales and to reduce the influence of differences in measurement units and numerical magnitude. These preprocessing steps were performed using information from the training data to avoid information leakage into the validation/test sets.

Table 1 presents a breakdown of potential outliers across variables in the formulation dataset, based on the IQR criterion. The table was used for data-quality assessment instead of removing the outliers. The number of outliers for each of the variables was quite different, with the Drug_PKa (18.95%) and SA-V (17.53%) variables showing the highest percentages of outliers. On the other hand, the Drug_TPSA and Drug_NHA contained very few potential outliers (1.64% each). It seems that these findings were due to the vast physicochemical and formulation diversity in the literature database and not data problems. Therefore, the values that were far off the normal range were marked but kept so as to keep the formulation variability consistent with the science, uphold the domain of the predictive models, and allow the proposed mechanism-informed framework to be applicable to different kinds of polymeric long-acting injectable formulations.

**Table 1:** Summary of outlier detection across dataset variables (IQR method).

Variable	Q1	Q3	IQR	Lower bound	Upper bound	Outlier count	Outlier (%)
LA/GA	0	1	1	−1.5	2.5	519	13.72
Polymer_MW	15,000	44,000	29,000	−28,500	87,500	298	7.88
Drug_Tm	206	262	56	122	346	487	12.87
Drug_Pka	10.36	11.75	1.39	8.27	13.84	717	18.95
Initial D/M ratio	0.11	1.00	0.89	−1.22	2.33	185	4.89
SA-V	2.89	155.56	152.67	−226.12	384.57	663	17.53
Drug_TPSA	78.29	221.29	143.00	−136.21	435.79	62	1.64
Drug_NHA	6	15	9	−7.5	28.5	62	1.64
Drug_LogP	2.42	3.74	1.32	0.44	5.71	432	11.42
Time	3.29	32.00	28.71	−39.78	75.07	311	8.22
T = 0.25	0.011	0.069	0.059	−0.077	0.157	157	4.15
T = 0.5	0.021	0.118	0.097	−0.125	0.265	195	5.15
T = 1.0	0.040	0.182	0.142	−0.173	0.395	188	4.97

The first box in Figure 1 is a visual presentation showing the spread of some major polymer, drug, and formulation descriptors before and after transformation. The main reason for the transformation, as you can see, was to put each variable on the same scale with an average around zero, with the variability being more or less comparable. However, despite the transformation, the characteristics of these distributions that include multimodality or a distribution of frequencies skewed either way, and the occurrence of discrete values have remained. These were variables like LA/GA and CL Ratio whose values were limited (in most cases, only two possible categories), so it makes more sense to treat them as categorical (in the case of the CL Ratio variable, maybe it could even be better treated as ordinal because it actually represents a ratio). At the same time, Polymer_MW and Drug_Tm variables displayed broad and quite distinct heterogeneous distributions reflecting the variety of formulation situations. Standardization is a good way to make comparisons between different parameters, but only through the method of comparing their values as numbers. Therefore, scale-dependent biased influence in further modeling or mechanistic analysis was effectively circumvented without losing the physicochemical signals that make these predictions and interpretations reliable and actionable.

**Figure 1:**
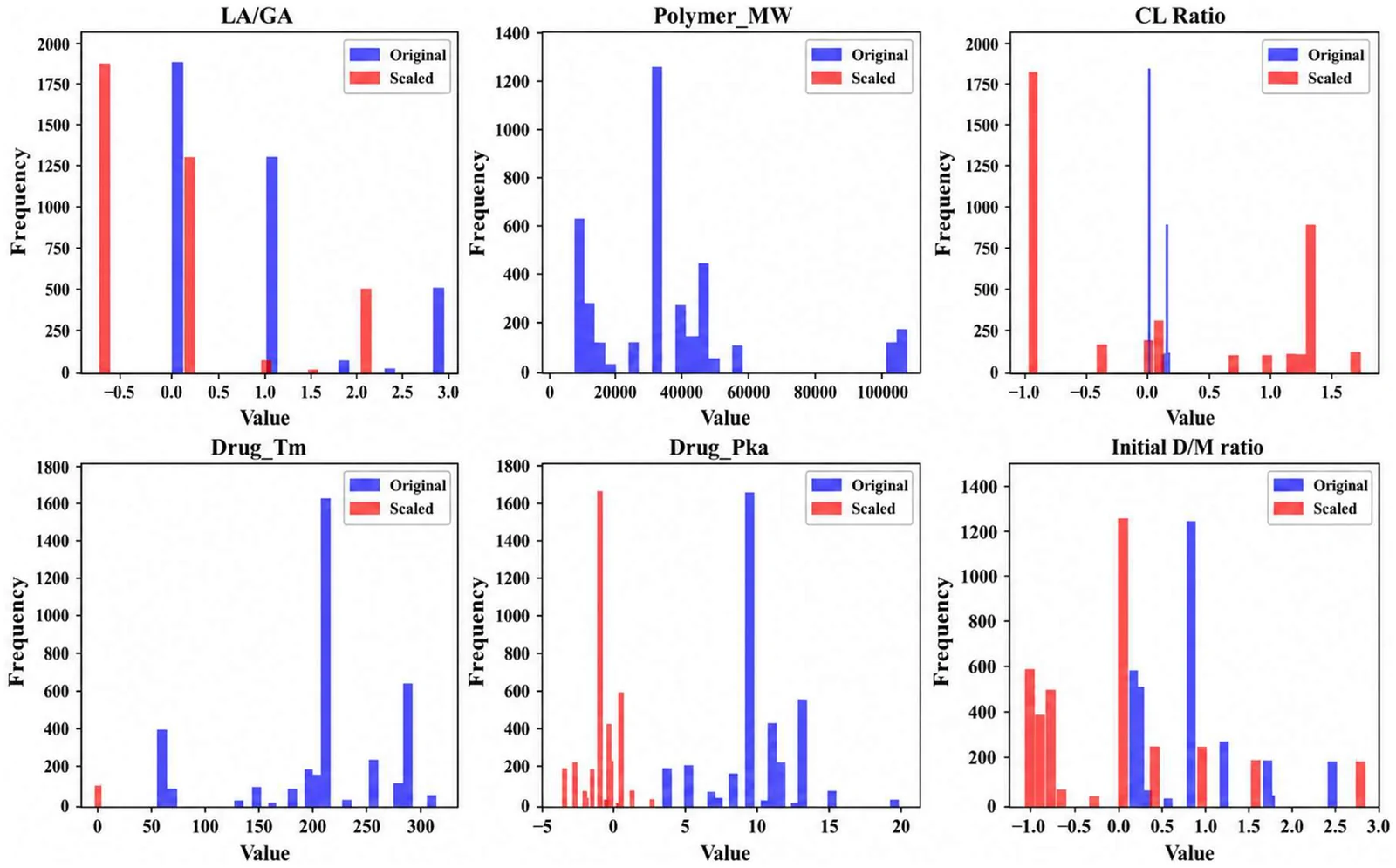
Distribution of representative features before and after standardization.

Figure 2 shows the relationships among 15 features with major influences on overall drug release. Overall release showed the strongest relationship with time (r = 0.49), indirectly indicating that, in cumulative drug release, time is the main governing factor. Other positive correlations of lower but still significant intensity were noted for the early-release variables (T = 1.0, T = 0.5, and T = 0.25), and they are in line with what was expected since the release mechanism should remain relatively consistent over time. Very high correlation values (r = 0.92, 0.97) between the time-related variables suggest that those early-release descriptors are indeed quite similar to the point where they could be called redundant. In fact, almost all variables related to the form and drug-polymer combination showed only minimal relationships with drug release, indicating that finding a certain degree of association does not guarantee it will lead to the correct variable for potential intervention. To achieve this identification, a combination of graph-building, mechanism-aware approaches, and effect estimation is necessary so that predictive associations can be separated from formulation effects of an intervention-oriented type.

**Figure 2:**
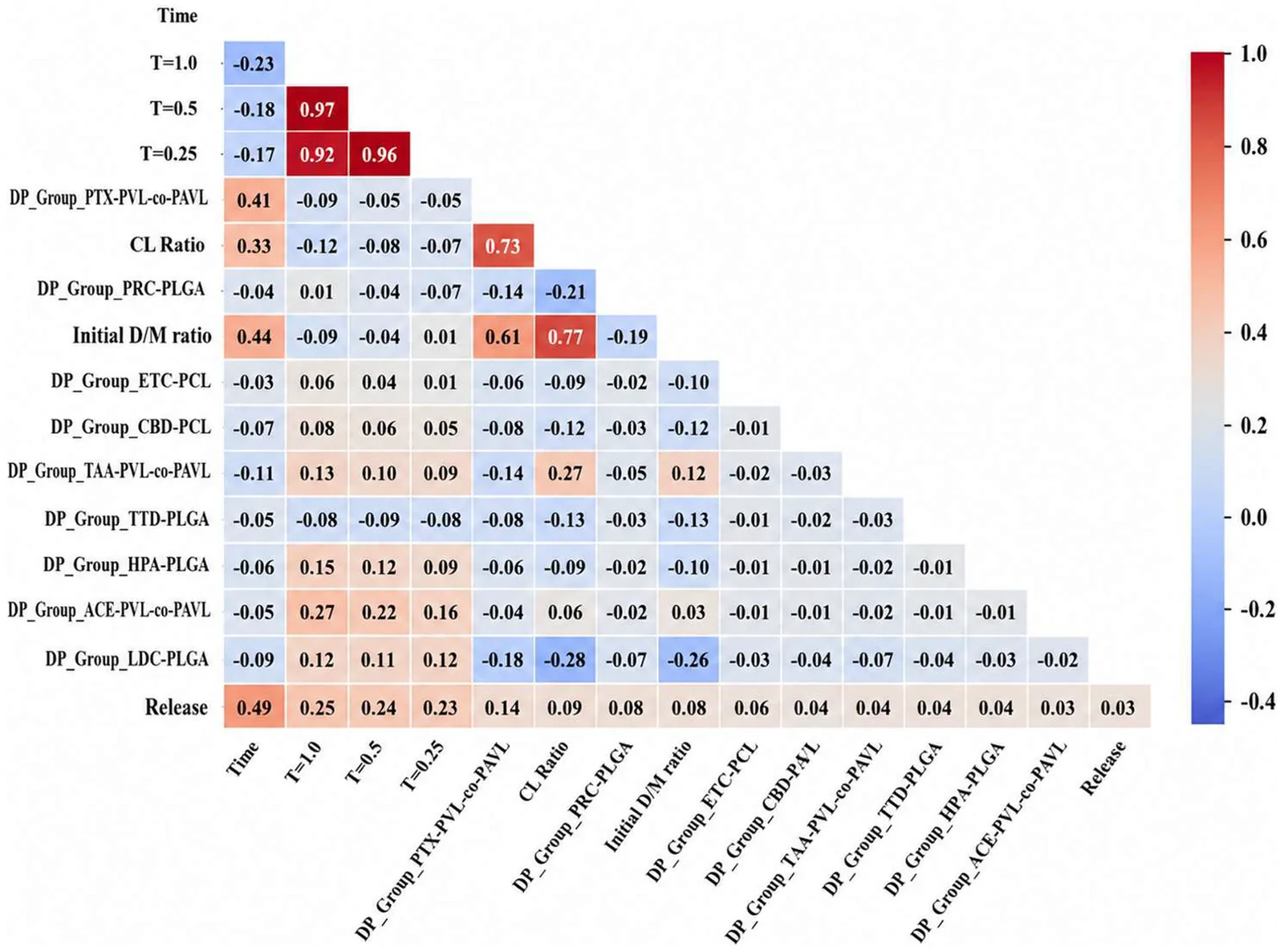
Correlation heatmap of the 15 features most strongly correlated with drug release.

Figure 3 ranks all preprocessed features according to their Pearson correlation with cumulative drug release, highlighting substantial differences in their statistical associations. *Time* exhibited the strongest positive correlation (r = 0.49), followed by the early-release variables (T = 1.0, T = 0.5, and T = 0.25), confirming the dominant influence of release progression on cumulative drug release. Several formulation descriptors, including CL Ratio and Initial D/M ratio, showed moderate positive correlations, whereas variables such as LA/GA and selected drug–polymer group indicators displayed weak negative associations. Notably, most physicochemical descriptors exhibited relatively small correlation coefficients, indicating that linear association alone provides limited insight into their influence on release behavior. These findings reinforce the need for complementary model-based interpretation and mechanism-informed effect estimation to distinguish predictive associations from variables with greater intervention relevance for formulation optimization.

**Figure 3:**
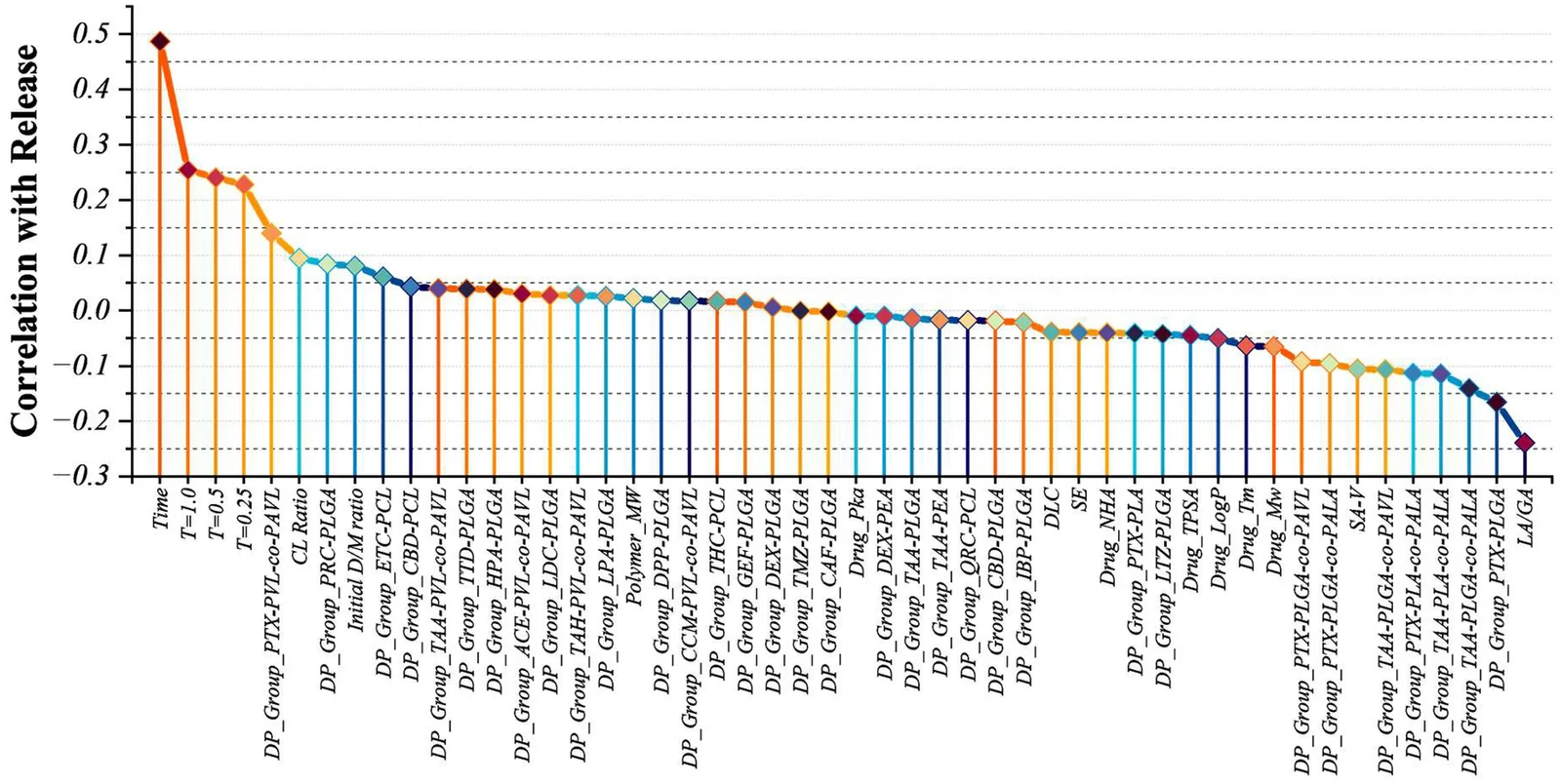
Correlation of all preprocessed features with drug release.

## 3 Methodology

The framework comprises four stages and a post-processing section, each running checkpointed in sequence, with each successive stage picking up the pickled outputs of the stage before it, producing artifacts (Excel tables, PDF figures, pkl state, etc.) so that exact re-execution without any loss of context can be possible. All stages use the fixed seed random_state = 123/np.random.seed(123); the row order is only shuffled once in Stage 2 and then shared to downstream stages using the same seed so as to ensure identical row ordering. Stage 3 here uses causal discovery via correlation-thresholding as well as mutual information-based scoring.

### 3.1 Stage 1. Preprocessing

Excel data loaded, row-shuffled (seed = 42), checked for missing values (replaced with medians), removed duplicates, and IQR outliers identified but not deleted (21). Data cleaning completed. Categorical features were transformed with one-hot encoding. Then, feature (target) separation was carried out (X = all columns except {Experimental_index, Release}, y = Release), and normalization was done with StandardScaler (22). Spearman/Pearson correlation of each feature of the dataset vs. Release was calculated, and the results were sorted (Algorithm 1).

Algorithm 1
df ← shuffle(read_excel(), seed=42)

df ← impute_median(df); df ← drop_duplicates(df)

outliers ← IQR_flag(df, k=1.5) # flagged only

X, y ← df.drop([index, Release]), df[Release]

X ← one_hot(X); X_scaled ← StandardScaler().fit_transform(X)

corr ← corr(X_scaled, y).sort_desc()

save(X_scaled, y, scaler, corr → step1_results.pkl)

### 3.2 Stage 2. Knowledge-based DAG

A directed graph is built manually from pharmaceutical domain knowledge (not learned) (23), e.g., drug/polymer property → DLC (drug loading) → Release; direct drug/polymer/formulation/time → Release; Temporal chain: Time → T = 0.25 → T = 0.5 → T = 1.0. If the graph is cyclic, the function nx.is_directed_acyclic_graph returns False, and a cycle (e.g., Drug_TPSA→Drug_pKa) triggers the edge removal automatically (Algorithm 2).

Algorithm 2
G ← DiGraph(); G.add_nodes(features+[Release])

G.add_edges([(Drug_LogP, DLC), (Polymer_MW, DLC), (LA/GA, DLC),

 (Drug_*, Release), (Polymer_*, Release),

 (Initial_D/M, DLC, SA-V, SE → Release),

 (Time→T0.25→T0.5→T1.0→Release)])

assert is_DAG(G); if not: remove_edge(cycle_edge)

save(G → dag.pkl)

The initial DAG was constructed by translating established pharmaceutical relationships into directed dependencies among formulation, physicochemical, experimental, and drug-release variables. The direction of each edge was assigned according to the presumed mechanism of influence rather than statistical association alone. Specifically, formulation and material properties were treated as upstream variables because they determine characteristics of the polymer matrix and drug environment; intermediate physicochemical or release-related properties were positioned downstream of these formulation factors; and cumulative drug release was treated as the final outcome. For example, polymer characteristics and formulation composition were connected toward variables representing the resulting release environment, which in turn were connected toward drug-release behavior. Experimental conditions were represented separately when they could influence the measured release response. This knowledge-based structure was subsequently used as a mechanistic prior for data-driven causal structure learning, allowing the learned relationships to be evaluated while retaining the pharmaceutical directionality considered biologically and physically plausible. Thus, the DAG does not simply represent correlations observed in the dataset; its initial edge directions reflect the expected order of influence derived from pharmaceutical knowledge.

### 3.3 Stage 3. Causal discovery

Two knowledge-driven plots are derived and compared to the knowledge DAG for Stage 2:

*Correlation plot:* Keep edge (i→j) only if it is highly and statistically significantly correlated (|Pearson r| > 0.15 and *p* < 0.01); the direction follows the variable with the stronger correlation with release.
*MI plot:* same orientation rule except that mutual_info_regression is used with the cut-off value of 0.05 (24).

Consensus graph keeps only edges that are present in ≥2 elements of {correlation, MI, knowledge}, and the edges are ranked by the number of agreements; Jaccard similarity is an estimate of the degree of overlap between three graphs in a pairwise comparison (Algorithm 3).

Algorithm 3
for i,j in variable_pairs:

 r, p ← pearsonr(X_i, X_j)

 if |r|>0.15 and p<0.01: orient_edge(i,j, by=|corr_with_Release|)

mi ← mutual_info_regression(X_i, X_j) per pair; same thresholding at mi>0.05

consensus ← {edge: score(edge)=[edge∈corr]+[edge∈mi]+[edge∈knowledge] ≥ 1}

### 3.4 Stage 4. Causal effect estimation

The treatment variable was chosen as the consensus-DAG direct cause of release with the highest |correlation| multiplied by (1 + unique_ratio) score; confounders are the overlap between treatment's and outcome's DAG parents (if there are fewer than 3 DAG parents, correlated variables, r > 0.15, are backfilled) (Algorithm 4). The four different estimators estimate the same (treatment, outcome, confounder) triple (25):

Algorithm 4
ATE_DoWhy ← CausalModel(T,Y,confounders).estimate_effect(“backdoor.linear_regression”)

ATE_DML ← EconML.LinearDML(model_y=RF, model_t=RF, cv=5).effect(X).mean()

ATE_CForest ← EconML.CausalForest(n_estimators=100).effect(X); CI ← effect_interval(*α*=.05)

ATE_LinDML ← Lasso-residualize(Y|conf), Lasso-residualize(T|conf) → Ridge(resid_T, resid_Y).coef

All four (DoWhy, DML, Causal Forest, Linear DML) have RandomForest/Lasso-based fallbacks if dowhy/econml are not installed. Per-confounder marginal contribution to ATE is computed by perturbing each confounder ±1 SD around its mean and re-residualizing.

### 3.5 Stage 5. Predictive modeling

70/15/15 train/validation/test split (train_test_split, seed 42, two-stage). Five regressors trained independently (26) (RandomForest, GradientBoosting, XGBoost, LightGBM, CatBoost; each ∼100 estimators, depth 5–10, random_state = 42). Metrics: R^2^, MAE, RMSE, MAPE (with ε-guard against division by zero and >1,000% cap) computed on train/val/test/all splits. Best model selected by highest Test R^2^, validated via 5-fold CV (Algorithm 5).

Algorithm 5
X_tr,X_va,X_te,y_tr,y_va,y_te ← split(X_scaled, y, 0.70/0.15/0.15, seed=42)

for m in [RF, GBR, XGB, LGBM, CatBoost]:

 m.fit(X_tr, y_tr)

 metrics[m] ← {R2, MAE, RMSE, MAPE} on {train, val, test, all}

best ← argmax(metrics[:, ‘Test_R2’])

cv_r2 ← cross_val_score(best, X_scaled, y, cv=5, scoring='r2')

### 3.6 Stage 6. Interpretability

The authors calculated three different kinds of importance scores for each feature, normalized these scores to the range [0, 1] to compare them easily: (i) the feature's absolute correlation with the target; (ii) TreeSHAP mean(|SHAP value|) (27) of the best model (if the SHAP estimation fails, use permutation importance instead); (iii) the per-confounder causal effect in Stage-4. They then did a pairwise Pearson correlation to find out how much the three standardized importance vectors agree or disagree among the correlational, model-based, and causal methods.

### 3.7 Stage 7. Counterfactual analysis

Baseline = per-feature median of X_scaled. Primary feature (Time, if present among top SHAP/causal features, else top-ranked feature) is swept across its empirical percentiles {0,10,25,50,75,90,100} with all other features held at baseline; predictions from the best model define the counterfactual release curve, with the P50 prediction as reference baseline (28). A secondary sweep perturbs the top 6 features individually by ±15%/±30% (heatmap of *Δ*release), and a joint multi-feature scenario grid (Time   ×   LA/GA × Drug_MW combinations) is evaluated for compound effects (Algorithm 6).

Algorithm 6
baseline ← median(X_scaled)

for p in [0,10,25,50,75,90,100]:

 x ← baseline.copy(); x[primary_feature] ← percentile(X_scaled[primary_feature], p)

 curve[p] ← best_model.predict(x)

for feat in top6_features, *Δ* in [−30,−15,0,15,30]%:

 x ← baseline.copy(); x[feat] *= (1+*Δ*)

 heatmap[feat][*Δ*] ← best_model.predict(x)−curve[50]

All Excel/pickle/figure outputs are numbered per stage (e.g., outputs/01_… through outputs/48_…, figures/01_… through figures/25_…), so any result in the paper can be traced to a specific saved artifact for reproducibility appendices.

The stages of the proposed framework are sequentially connected, with the output of each stage providing the basis for the subsequent analysis. First, the knowledge-informed DAG establishes the hypothesized directional relationships among formulation, physicochemical, experimental, and release variables, while causal discovery is used to refine these relationships using the observed data. The resulting causal structure is then used to identify potential treatment variables and their relevant outcome pathways for causal effect estimation. ATE, CATE, and individual treatment effects quantify the estimated changes in drug-release behavior associated with interventions on these variables under different conditions. These estimated effects subsequently provide the basis for counterfactual analysis, in which alternative formulation settings are evaluated by asking how the predicted release outcome would change if a selected formulation variable were modified while other relevant conditions were held constant. Thus, the causal graph defines the structural context, causal effect estimation quantifies intervention effects, and counterfactual analysis translates these effects into virtual formulation scenarios for formulation prioritization.

## 4 Result and discussion

### 4.1 Mechanism-informed causal structure construction

Figure 4 shows the pharmaceutical knowledge-based DAG, which is basically the structural backbone of the new mechanism-based design framework being described. The graph captures the assumed interrelations among various drug properties, polymer properties, formulation variables, the time factor (temporal), measurement, and accumulated drug release, which are based on well-known pharmaceutical concepts. The nodes in the graph, especially DLC, early-release results, and important physicochemical descriptors, act as a sort of middlemen between the formulation characteristics and the end release output. The temporal variables are shown in the graph through a time axis as an explicit element depicting when the various stages of drug release happen. While it is tempting to derive the relationship solely by looking at the statistical patterns, the DAG uses the knowledge of domain experts to provide a boundary for the estimation of effects and virtual interventions. This structured representation provides a transparent and mechanistically interpretable basis for distinguishing predictive associations from intervention-oriented relationships within polymeric long-acting injectable formulations.

**Figure 4:**
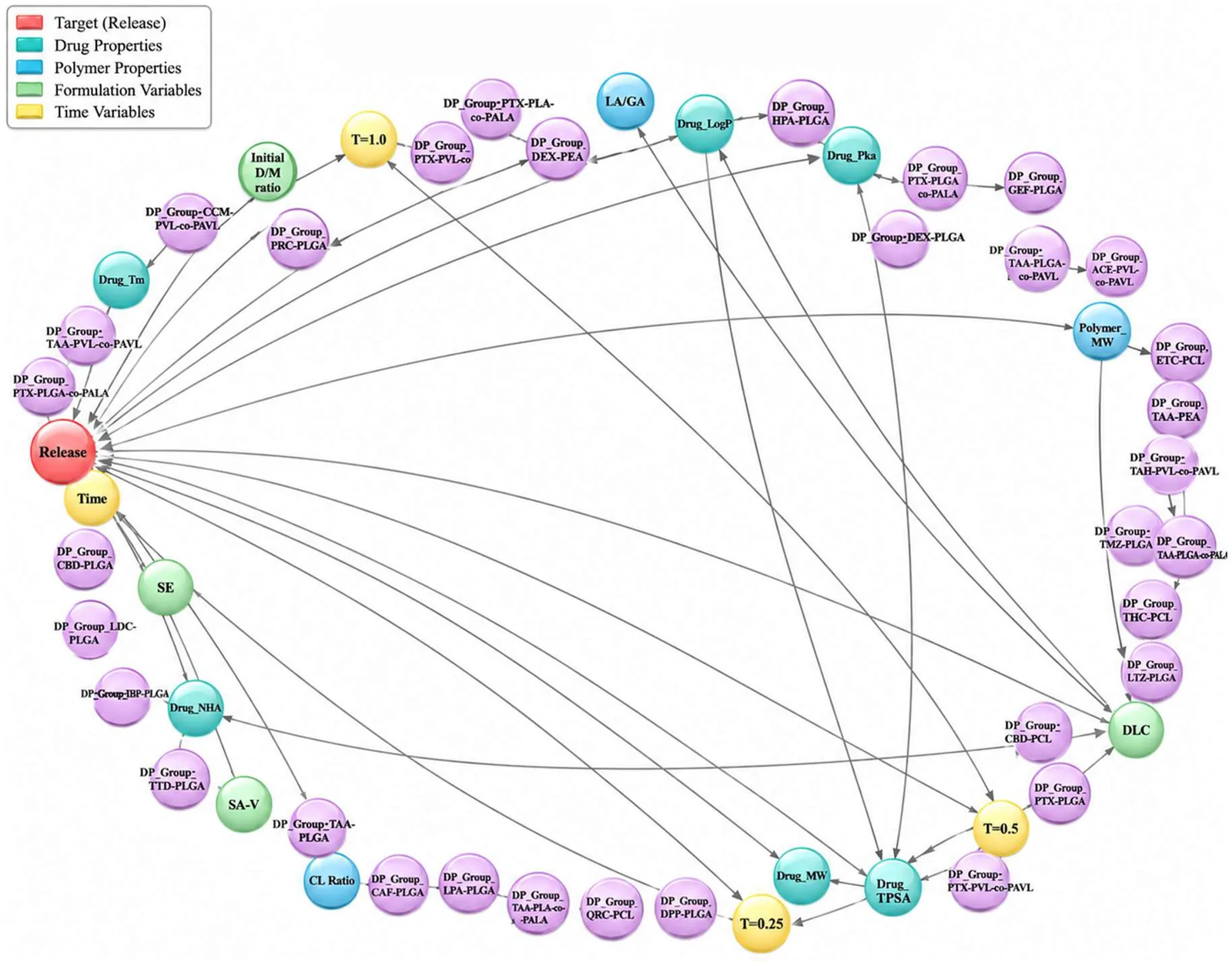
Full causal DAG for drug release prediction (pharmaceutical knowledge-based).

Figure 5 shows a simplified version of the knowledge-driven directed acyclic graph, focusing only on the variables that have a direct effect on the total drug release. The simplified figure therefore captures the target variable's dependencies while also revealing that these dependencies are indirect when the full network is considered. The features of the drug, polymer, formulation parameters, and time variables are the main contributors directly to the release of the drug, and this demonstrates that controlled drug release is a multifactor scenario. The diagram highlights that release behavior is mainly due to several combined effects of physicochemical characteristics and the design of the formulation in the form of a product rather than being due to any one of them. This simplified layout of the structure will then serve as a base for the later estimation of mechanism-informed effects and virtual intervention analyses by clearly laying out the hypothesized dependency graph.

**Figure 5:**
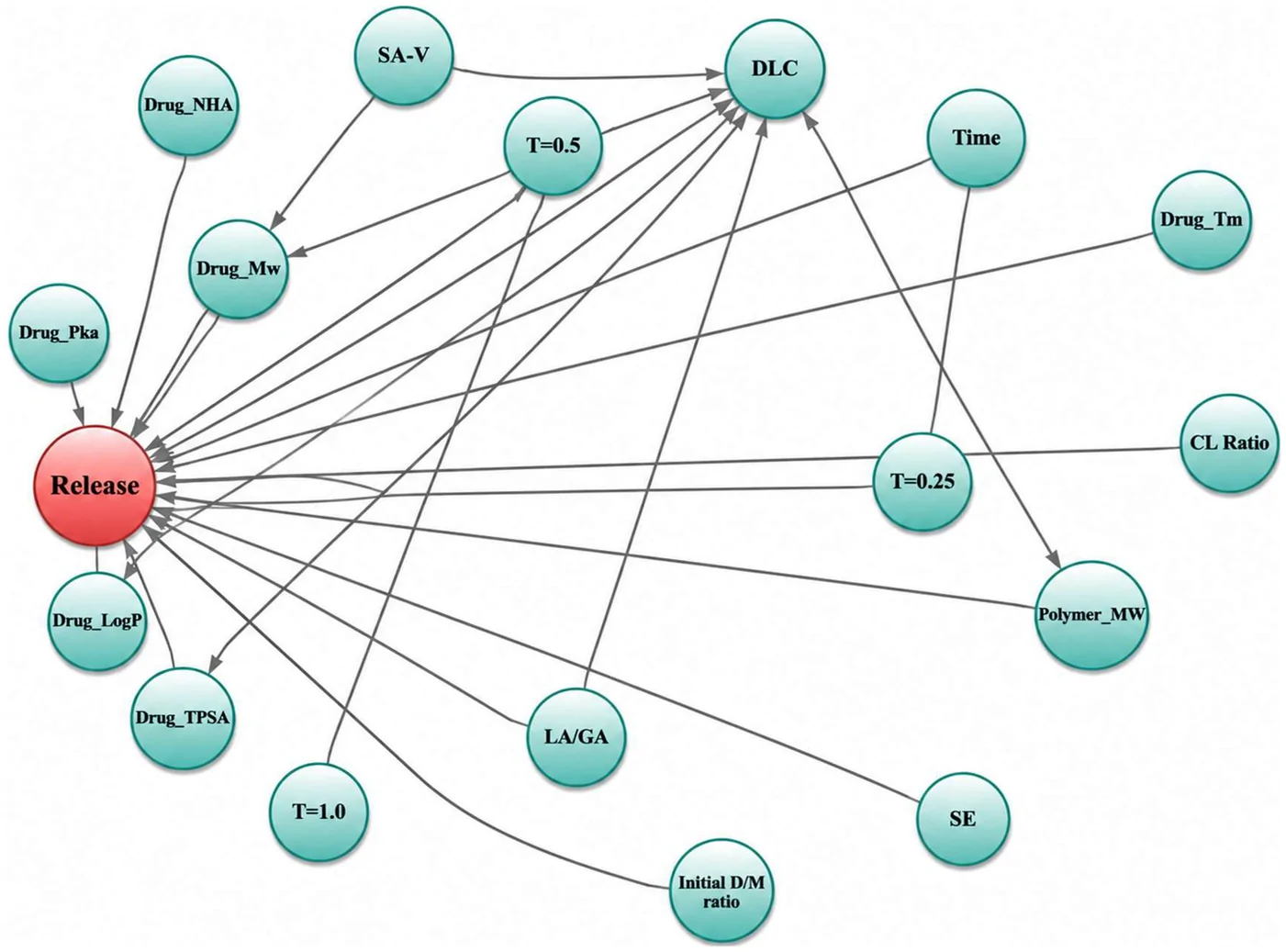
Direct causes of drug release (simplified DAG).

Figure 6 presents proposed links between drug release features of drugs, as described by their physicochemical properties, and a release-related outcome in the novel mechanism-informed drug release model. Molecular properties of drugs such as weight, solubility in fats (lipophilicity), solidification temperature (melting point), ionization constant (pKa), topological polar surface area, and heteroatom count are linked through intermediate associations to reach the release profile. DLC also comes in as another intermediate node; it is a key indicator of a drug's molecular traits that may be reflected in a particular formulation performance. The model instead treats drug properties, which shape release as an outcome, as interdependent rather than assuming them as separate. This kind of visual depiction of the underlying causal connections would facilitate biological understanding and enable further modeling of effects of interventions by the means proposed in the graphical model.

**Figure 6:**
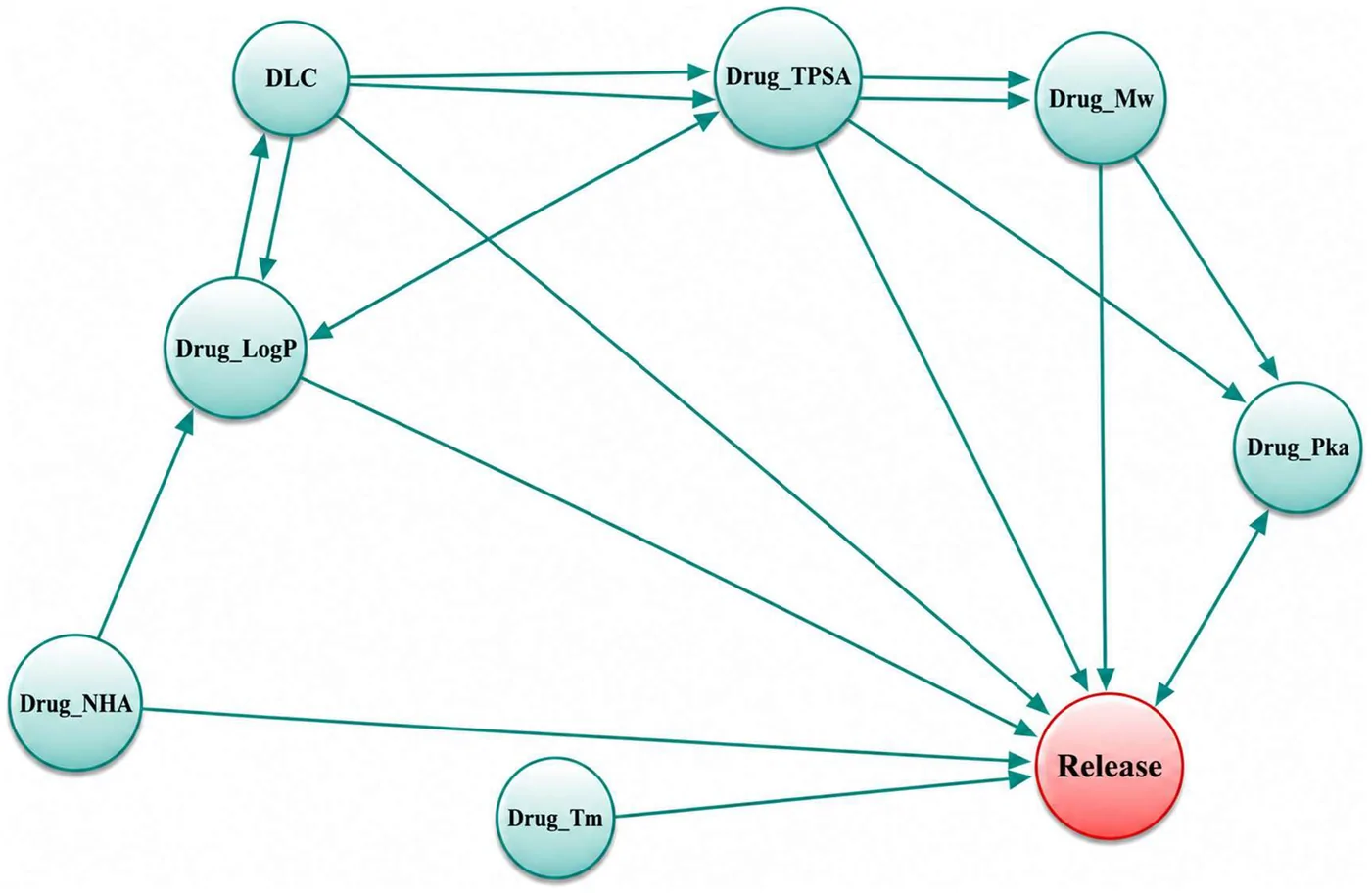
Causal pathway from drug properties to release.

Figure 7 illustrates the interdependent factors governing drug release and polymer properties. A visual analysis of the illustration brings out how a polymer's molecular weight, LA/GA ratio, CL Ratio, and DLC are not isolated, but rather linked variables that together control the rate and extent of a drug's release from a polymer. Drug loading capacity, as the central hub, shows the linkage between polymer formulation and the molecular weight of the polymer and, finally, release behavior, which would be the ultimate result. It is likely that changes in formulation properties can affect release via different mechanisms. Even though the flow-chart represents only pharmaceutical theories rather than those established via experiments, it does offer a lucid view of drug degradation mechanisms that could result in the liberation of the therapeutic agent from polymer vehicles. This pathway provides a basis to evaluate intervention-estimated effects in the defined context of a mechanistically plausible framework.

**Figure 7:**
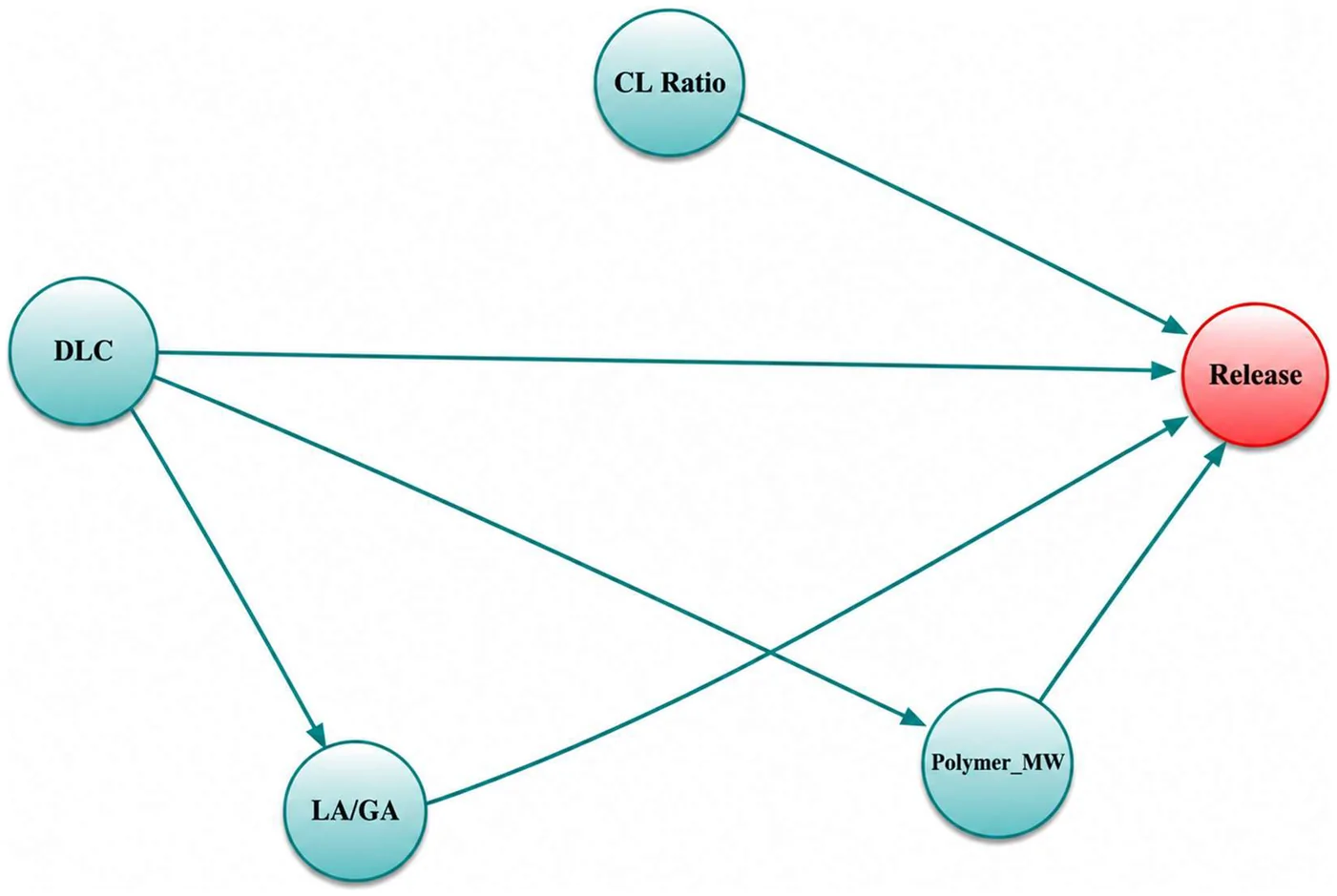
Causal pathway from polymer properties to release.

Figure 8 shows the time part of the suggested graphical model that links the early time point observations (T = 0.25, T = 0.5, and T = 1.0) with the drug release progression of time and the cumulative drug release. The strong sequential dependencies between the early release measurements reveal how the release characteristics progress over time. At the same time, the direct connections from these measurements to the release node show the predictive influence each measurement has on the final outcome. Instead of viewing the data points as independent variables, this temporal design explicitly considers a sequence of drug release evolutions. As a result, the method retains dependencies over time found in controlled-release systems. It also provides a clearer mechanism-based interpretation, which makes it much better for prediction and intervention analyses than conventional ML models.

**Figure 8:**
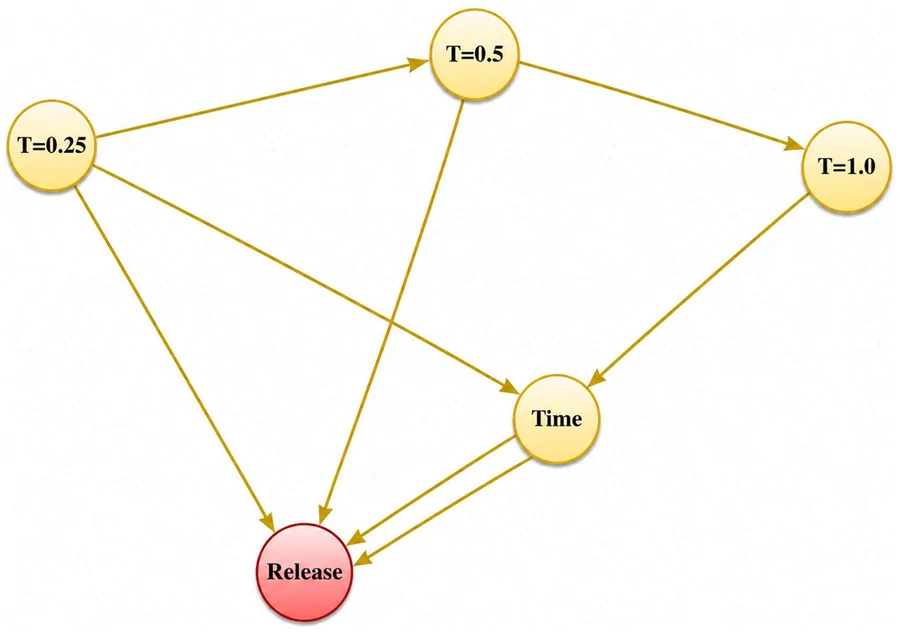
Causal pathway from time to release.

Figure 9 shows the four graph construction methods used in the mechanism-informed framework: correlation, mutual information, a pharmaceutical knowledge source, and the final consensus graph. The correlation-based network (122 edges) displays linear statistical relationships. By contrast, the MI-based network (205 edges) not only identifies linear dependencies but also nonlinear ones, resulting in the highest level of connectivity. The pharmaceutical knowledge-based DAG, on the other hand, is far less dense (29 edges), since it removes all relationships that lack support from formulation mechanisms and keeps a DAG only as long as it does not create loops. Combining all three graph sources, the consensus graph (100 edges) reflects the most balanced empirical and domain knowledge network while still maintaining biologically plausible relationships by removing data-driven associations solely based on the graph structure. This is a great step forward from using single sources for constructing the underlying network to combining them in a consensus fashion while still keeping most of the relationships based solely on biological mechanisms.

**Figure 9:**
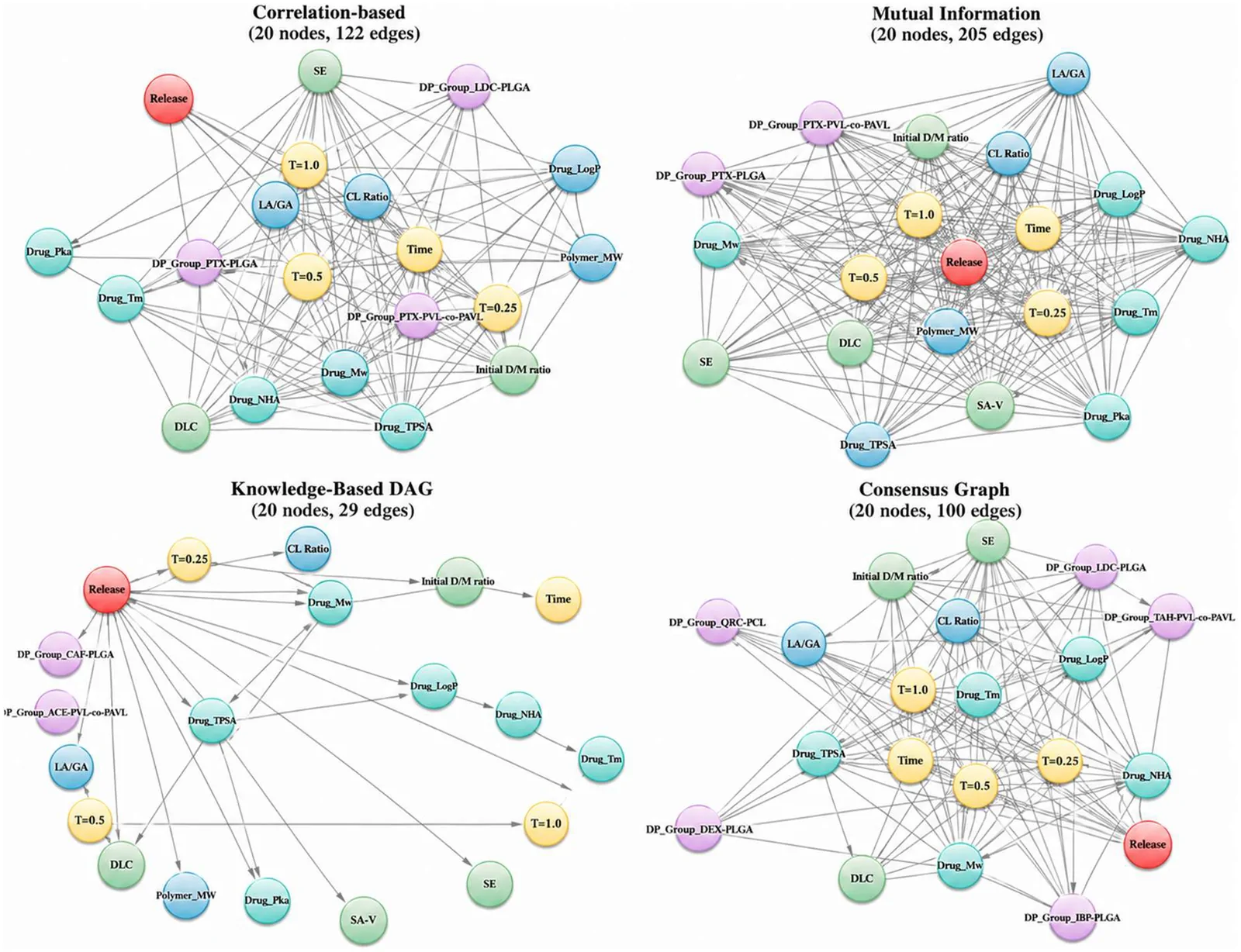
Comparison of causal discovery methods.

### 4.2 Predictive performance of ML models

Table 2 shows the comparison of predictive performance results for the five ensemble learning algorithms for cumulative drug release prediction. XGBoost achieved the best prediction accuracy, with a test R^2^ of 0.9774, the highest score, the smallest RMSE (0.0491), and the smallest MAE (0.0339). Moreover, it consistently gave good results in all datasets: training, validation, and test. Gradient Boosting and LightGBM delivered comparably good results. Apart from CatBoost, all the other methods performed with high predictive accuracy, with CatBoost having the lowest. The performance differences that occur over data partitions are relatively small, indicating that the behavior of the model is fairly stable and there is little evidence of overfitting. Because of its best performance on the validation set, XGBoost was chosen for further interpretability, mechanism-effect estimation, and virtual intervention study analysis, whereas the independent test set was kept for the final performance check.

**Table 2:** Performance metrics of ML models across train, validation, test, and full datasets.

	Models				
Metric	XGBoost	GradientBoosting	LightGBM	RandomForest	CatBoost
Train R^2^	0.9919	0.9856	0.9829	0.9852	0.9482
Train MAE	0.0213	0.0280	0.0303	0.0263	0.0551
Train RMSE	0.0295	0.0394	0.0429	0.0400	0.0747
Train MAPE (%)	23.36	25.19	27.25	10.38	34.76
Val R^2^	0.9788	0.9691	0.9684	0.9645	0.9357
Val MAE	0.0327	0.0393	0.0412	0.0409	0.0621
Val RMSE	0.0487	0.0588	0.0594	0.0630	0.0848
Val MAPE (%)	26.46	29.31	30.20	16.11	37.50
Test R^2^	0.9774	0.9711	0.9705	0.9641	0.9337
Test MAE	0.0339	0.0390	0.0401	0.0418	0.0622
Test RMSE	0.0491	0.0555	0.0562	0.0619	0.0842
Test MAPE (%)	26.03	26.81	29.55	14.64	35.23
All R^2^	0.9877	0.9809	0.9788	0.9788	0.9441
All MAE	0.0249	0.0314	0.0334	0.0308	0.0572
All RMSE	0.0364	0.0454	0.0479	0.0478	0.0778
All MAPE (%)	24.23	26.05	28.03	11.88	35.24

Five gradient-boosting and ensemble-based models were trained to predict cumulative drug release, evaluated via R^2^, mean absolute error (MAE), root mean squared error (RMSE), and mean absolute percentage error (MAPE) across training, validation, test, and combined splits. XGBoost achieved the highest test-set performance (R^2^ = 0.9774, RMSE = 0.0491) with the smallest train–test generalization gap, and was selected as the primary model for downstream interpretability and counterfactual analysis. CatBoost showed the weakest and least generalizable performance (test R^2^ = 0.9337).

XGBoost's predictive performance is also relevant from a formulation-development perspective. The RMSE of 0.0491 and MAE of 0.0339 represent relatively small prediction deviations on the normalized cumulative drug-release scale used for model development. Together with the test R^2^ of 0.9774, these results indicate that the model can reproduce the observed variation in cumulative drug release with high fidelity. This predictive performance supports using the model to compare formulation scenarios and screen candidate interventions before experimental testing. However, practical acceptability should ultimately be evaluated against experimental variability and the original measurement scale of cumulative drug release.

Figure 10 compares the XGBoost model's predictions to the experimentally determined drug release data of the independent test set. The majority of data points lie very tightly within the error bar limits (which in this plot corresponds to the 1:1 line), showing that the predicted drug release profile closely follows the actual one over the entire measurement range. This observation coincides with the high predictive abilities of the model, as evidenced by performance metrics given in Table 2 (test R^2^ = 0.9774, RMSE = 0.0491), which further indicate the model's ability to correctly map complicated nonlinear relationships controlling the drug release mechanism. Prediction errors are still minor when dealing with low, intermediate, and high release scenarios, although a few observations are significantly off the line, which may correspond to formulations whose features remain partially undetected by the model. The lack of a constant trend of underprediction or overprediction suggests the model is well calibrated overall, thus validating its predictions in the range. This finding demonstrates that XGBoost could give an accurate prediction, which could be helpful for mechanism-driven understanding, effects estimation, and formulation virtual studies in this paper.

**Figure 10:**
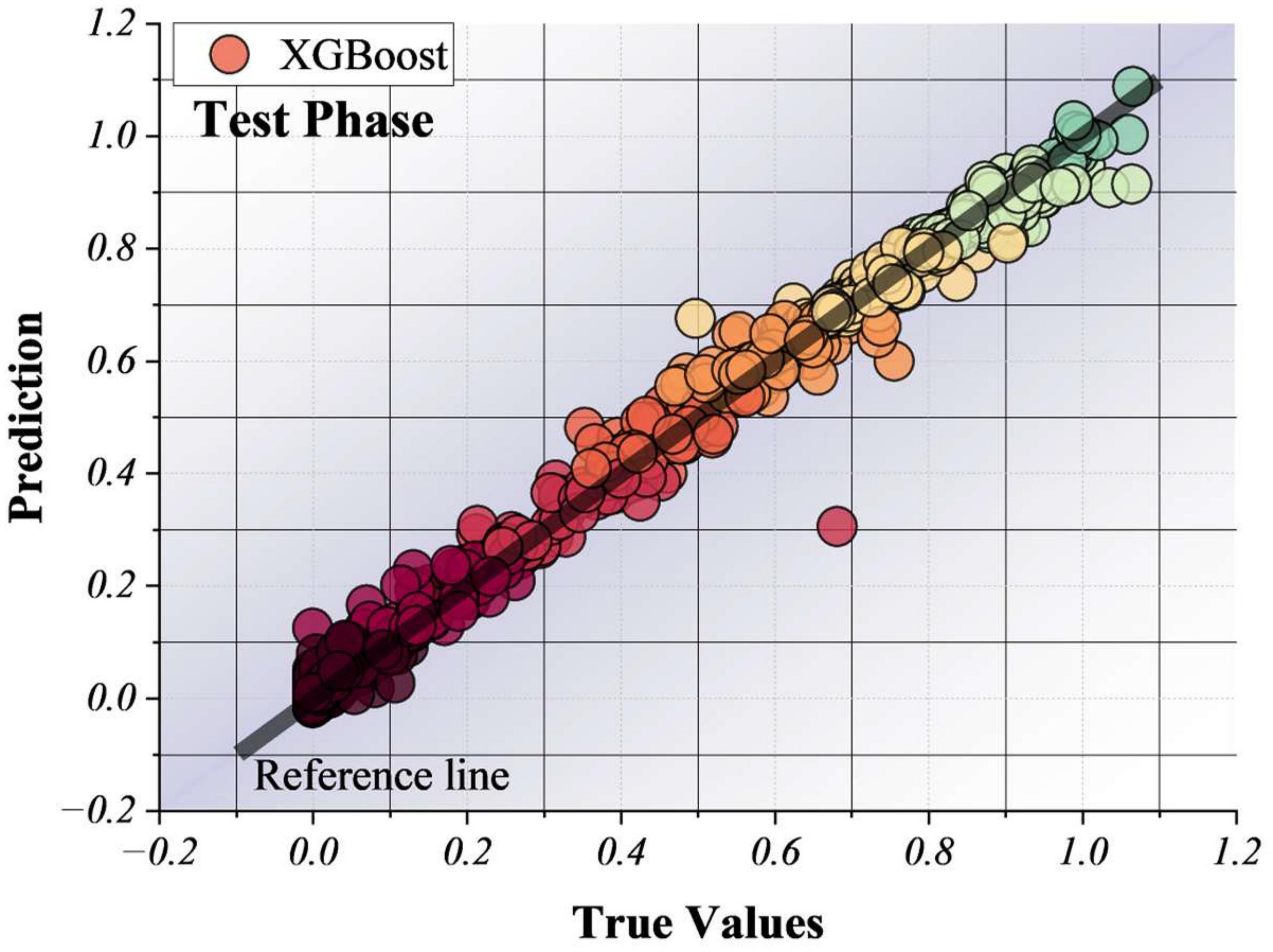
Predicted vs. actual drug release for the XGBoost model (test set).

### 4.3 Causal effect estimation and intervention analysis

Table 3 shows selected intervention scenarios where model-based release predictions were modified by jointly changing the release time, the LA/GA composition, and the molecular weight of the active compound, while the results of the prediction were evaluated by the trained ML model. The predicted release was low, at 0.212, in fact, in the most unfavorable configuration, which was also the first to appear. Then, the prediction went up to 0.917 in the most highly advanced scenario, while the baseline formulation resulted in an intermediate prediction of 0.442. These findings show that varying combinations of formulation and experimental parameters may lead to greatly different predicted release profiles. This approach does not aim at establishing cause-and-effect relationships; instead, these simulations offer a mechanistically informed virtual experimentation platform that allows exploration of potential modification(s) of the formulation before laboratory validation, thus supporting the development of more efficient formulation screening.

**Table 3:** Multi-feature counterfactual scenarios for predicted drug release.

Scenario	Time	LA/GA	Drug_Mw	Predicted release
T0.25_LA0_MW-0.5	0.25	0	−0.5	0.212
T0.5_LA1_MW0 (baseline)	0.50	1	0	0.442
T0.75_LA2_MW0.5	0.75	2	0.5	0.802
T0.9_LA3_MW1.0	0.90	3	1.0	0.917

Figure 11 summarizes the estimated effect of release time on cumulative drug release using four complementary intervention-effect estimators and the corresponding distribution of conditional treatment effects. Different intervention-effect estimators show the same direction of the effect of time on drug release, while their magnitude varies. The highest ATE was estimated by Causal Forest (0.3790), while EconML-DML estimated a much lower value, only 0.2658. DoWhy (0.1866) and Linear DML (0.1860) produced more conservative estimates. These magnitude differences are expected because the estimators rely on different modeling assumptions and estimation strategies. In particular, Causal Forest captures nonlinear and heterogeneous treatment effects, whereas Linear DML imposes a more restrictive linear functional form. EconML-DML uses a doubly robust, nuisance-model-based estimation strategy, while the DoWhy estimate depends on the specified causal model and identification procedure. Consequently, the estimators can agree on the direction of an intervention while producing different effect magnitudes because they represent the underlying treatment–outcome relationship differently. The consistent positive direction across all four estimators therefore provides stronger evidence for the direction of the estimated intervention effect than for any single numerical magnitude.

**Figure 11:**
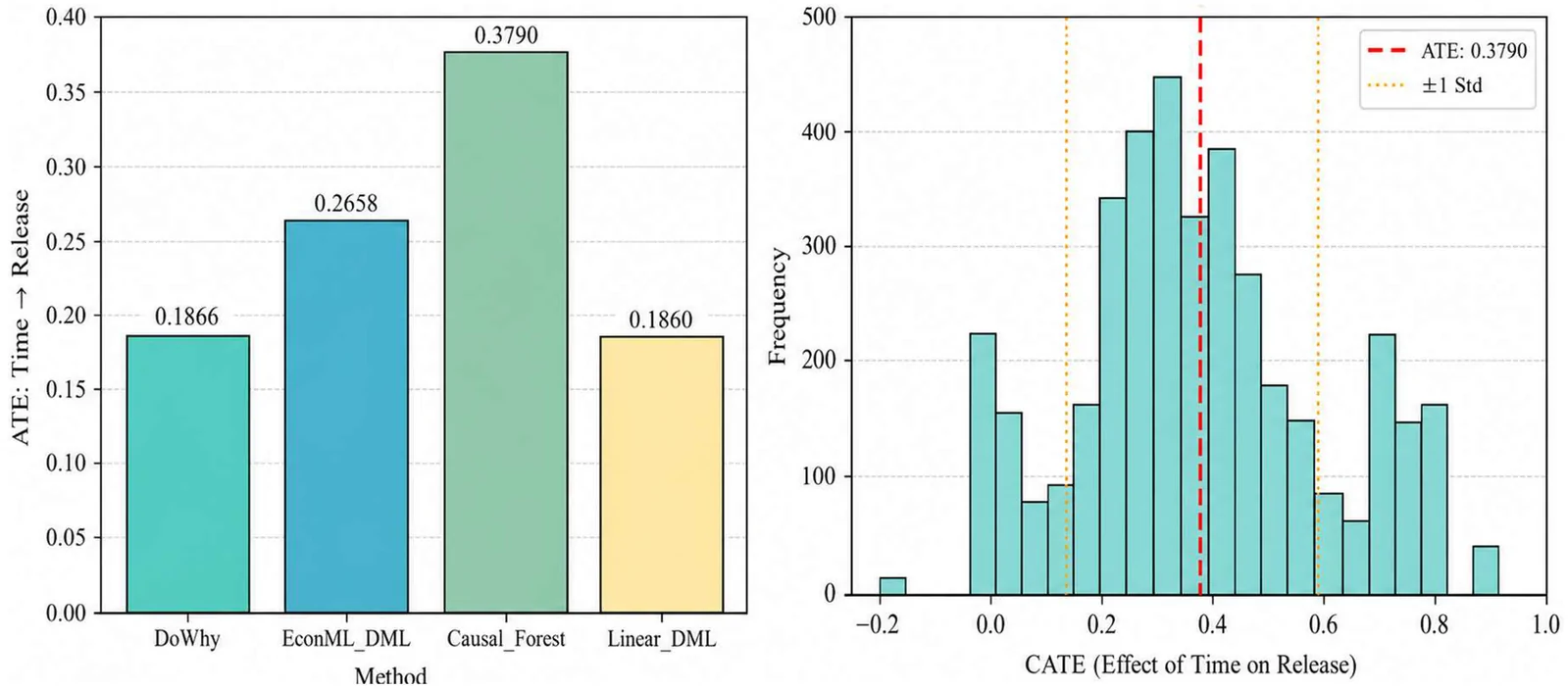
Causal effect estimation of release time on drug release.

The overall similarity in the direction of the estimated effects suggests that release time is positively associated with predicted drug release under the mechanism-informed graph assumptions. However, the variation from 0.1860 to 0.3790 indicates that the magnitude should be interpreted as estimator-dependent rather than as a single definitive intervention effect. A histogram of the CATE highlights the large variation among different formulations, as it is the case here that almost all effects center at an ATE but still stretch into a wide range of values. The fact that the same factor may affect different formulations in different ways clearly illustrates that there is no single influence of release time applicable across all polymer-drug systems. Thus, taken together, the results point to the significant value of formulation-dependent effect estimation.

### 4.4 Predictive vs. intervention-oriented feature importance

Table 4 gives a picture of the level of agreement between association-based, model-based (SHAP), and intervention-oriented feature importance measures. Correlation analysis and SHAP measures have a strong level of agreement, with a correlation coefficient of 0.761, suggesting that the predictors that were strongly associated with drug delivery have generally been given the highest importance in predictive models. On the other hand, the intervention effect estimates showed weak agreement with the correlation-based importance (r = 0.132) and almost no correlation with SHAP importance rankings (r = 0.021). This divergence can arise because predictive importance and intervention effects represent fundamentally different quantities. Correlation measures marginal statistical association, whereas SHAP importance quantifies a feature's contribution to the fitted ML model's predictions. In contrast, intervention effects estimate how the predicted outcome changes when a variable is deliberately modified within the specified causal structure. Nonlinear interactions, mediation, and dependencies among formulation variables can therefore allow a variable to contribute strongly to prediction without producing a correspondingly large change under intervention. Conversely, a variable with modest marginal or SHAP importance may have a substantial intervention effect when its modification propagates through relevant pathways or interacts with other formulation conditions. This implies that factors mainly influencing the prediction outcome may be completely different from those most effectively predicted when performing hypothetical interventions. This difference justifies the main argument for developing the framework, as it separates the predictive relevance from an analysis that is intervention-oriented.

**Table 4:** Correlation between feature-importance ranking methods.

Analysis	Correlation	SHAP	Causal effect
Correlation	1	0.761	0.132
SHAP	0.761	1	−0.021
Causal Effect	0.132	−0.021	1

Figure 12 reveals that feature rankings generated through traditional correlation analysis and SHAP-based model interpretation are alike yet distinct. Both feature sets agree that time is the leading driver for cumulative drug release by a wide margin, followed by the variables measuring early release (T = 1.0, T = 0.5, and T = 0.25), which, together with time, underscore the decisive role of a time factor in predicting drug release. Nonetheless, many formulation and physicochemical descriptors were ranked differently in the two analysis methods. In a couple of cases, namely SA-V, CL Ratio, and Initial D/M ratio, these descriptors were found in traditional correlation analysis to have a larger effect than in SHAP, while in the case of SHAP it turned out that, after accounting for the joint contribution of features within the fitted nonlinear model, it placed relatively less, and more evenly, importance on minor variables.

**Figure 12:**
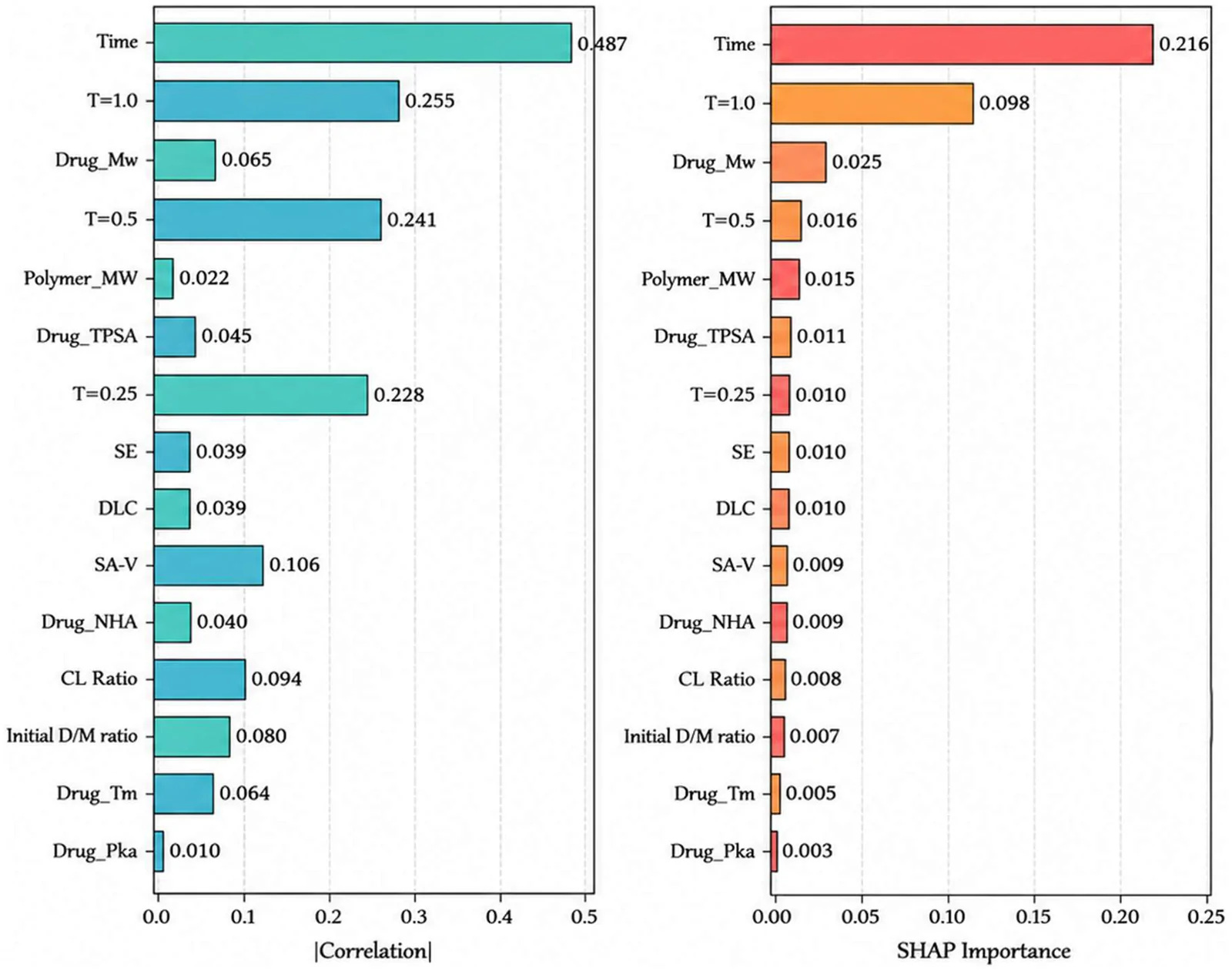
Comparison of feature importance rankings by correlation and SHAP value.

Such differences are expected because correlation does not account for the full multivariate structure learned by the model, whereas SHAP attributes prediction contributions within that structure. This situation highlights the differing ways in which statistical association and model-derived importance reflect on the prediction process. Therefore, correlation analysis cannot fully explain model behavior. This leads to the rationale for further comparisons through estimating effect sizes for interventions, which separates being predictors from being important variables for formulation optimization.

Figure 13 displays the rankings of feature SHAP importance in terms of average contribution to a single model's predictions globally for all XGBoost examples. Time has been determined as the leading factor (mean |SHAP| = 0.216) with a very significant difference compared to the rest of the variables, pointing to the fact that temporal development is, by far, the determining factor for cumulative drug release. Among the other parameters, the first-release measurement at T = 1.0 ranked second in importance (0.098); Drug_MW, Polymer_MW, Drug_TPSA, and T = 0.5 had a somewhat higher impact, though much lower than the leading features. Most formulation aspects, such as DLC, SA-V, CL Ratio, and Initial D/M ratio, had little impact on the model's predictions relative to the dominant temporal variables, after accounting for the joint and nonlinear relationships represented by the model. The SHAP values of the other features fall considerably below those of the first few; this means the prediction is governed by a few dominant features, not a uniform distribution of inputs. Although model behavior is well explained by SHAP, these importance measures do not reflect the impact a change (intervention) would have on a variable but rather the contribution they make to predictions, which is why these measures are compared with the effects of mechanism-informed estimation in a subsequent step, as suggested in the framework.

**Figure 13:**
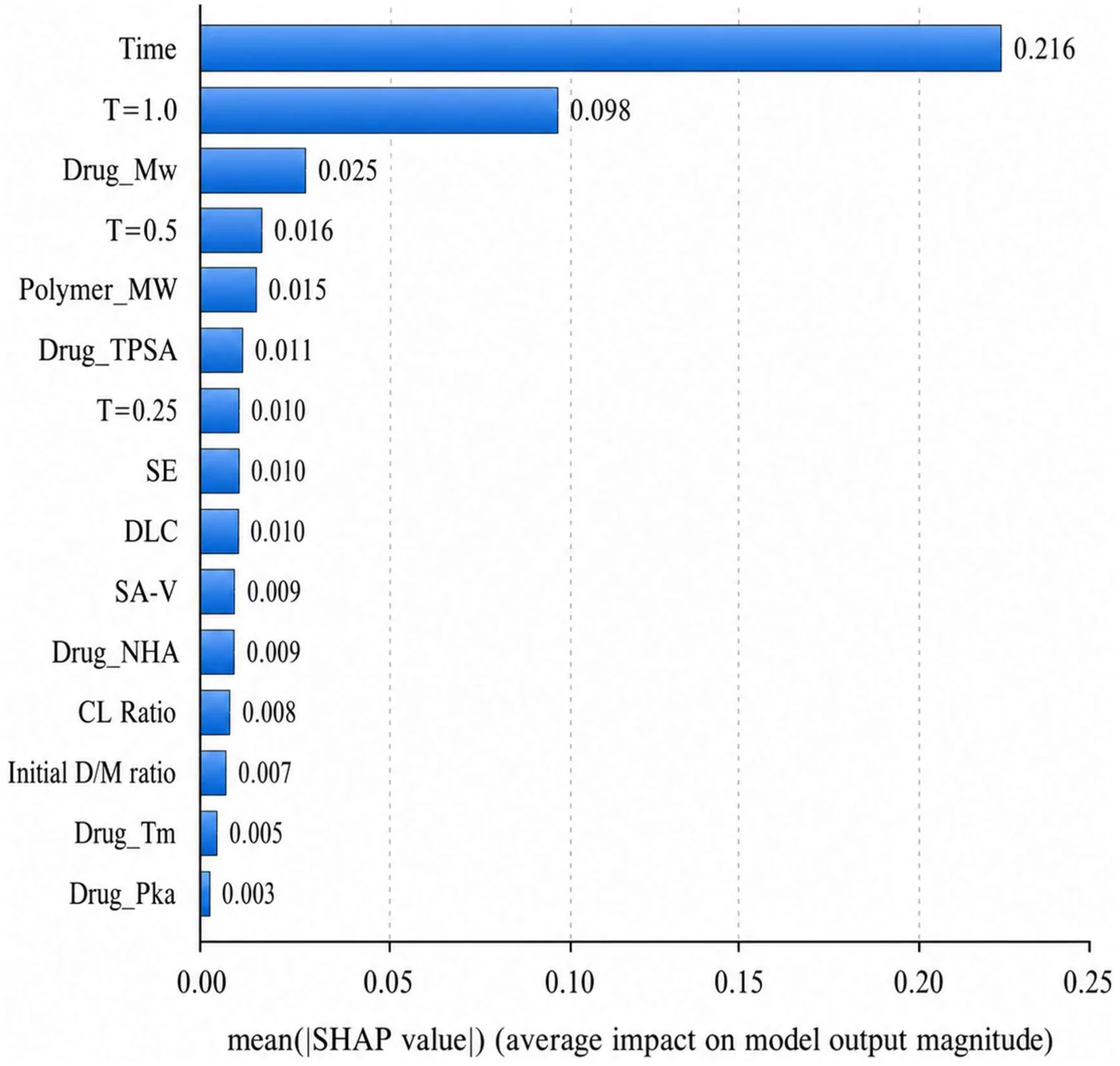
SHAP feature importance ranking (XGBoost model).

Figure 14 exhibits the SHAP beeswarm plot representing changes in both magnitude and direction of each feature's contribution to the XGBoost outputs as it goes through all instances of formulation. The SHAP ranks at the global level corroborate that time has the most significant effect; higher levels of time (red points) mostly correspond to large SHAP values, which implies that a higher value of Drug release will be predicted. On the other hand, a lower level of time (blue points) will significantly reduce the model output. T = 1.0 (early- release variable) showed a very similar pattern except that the magnitude of its impact was less. On the other hand, the variablesDrug_MW, Polymer_MW, Drug_TPSA, and DLC had more restricted SHAP distributions around zero, indicating their contributions were less pronounced and dependent on the formulation composition. The fact that the positive and negative SHAP values for the descriptors overlap means that their impact depends on how the variables that constitute formulations interact with each other rather than following a purely monotonic trend. The beeswarm plot shows that the model reflects nonlinear formulation dependencies, but the prediction is still largely driven by temporal information. These findings serve as a complement to the importance rankings by illustrating how feature effects may vary across different polymeric long-acting injectable formulation instances; also, they provide interpretable information at the individual model instance level.

**Figure 14:**
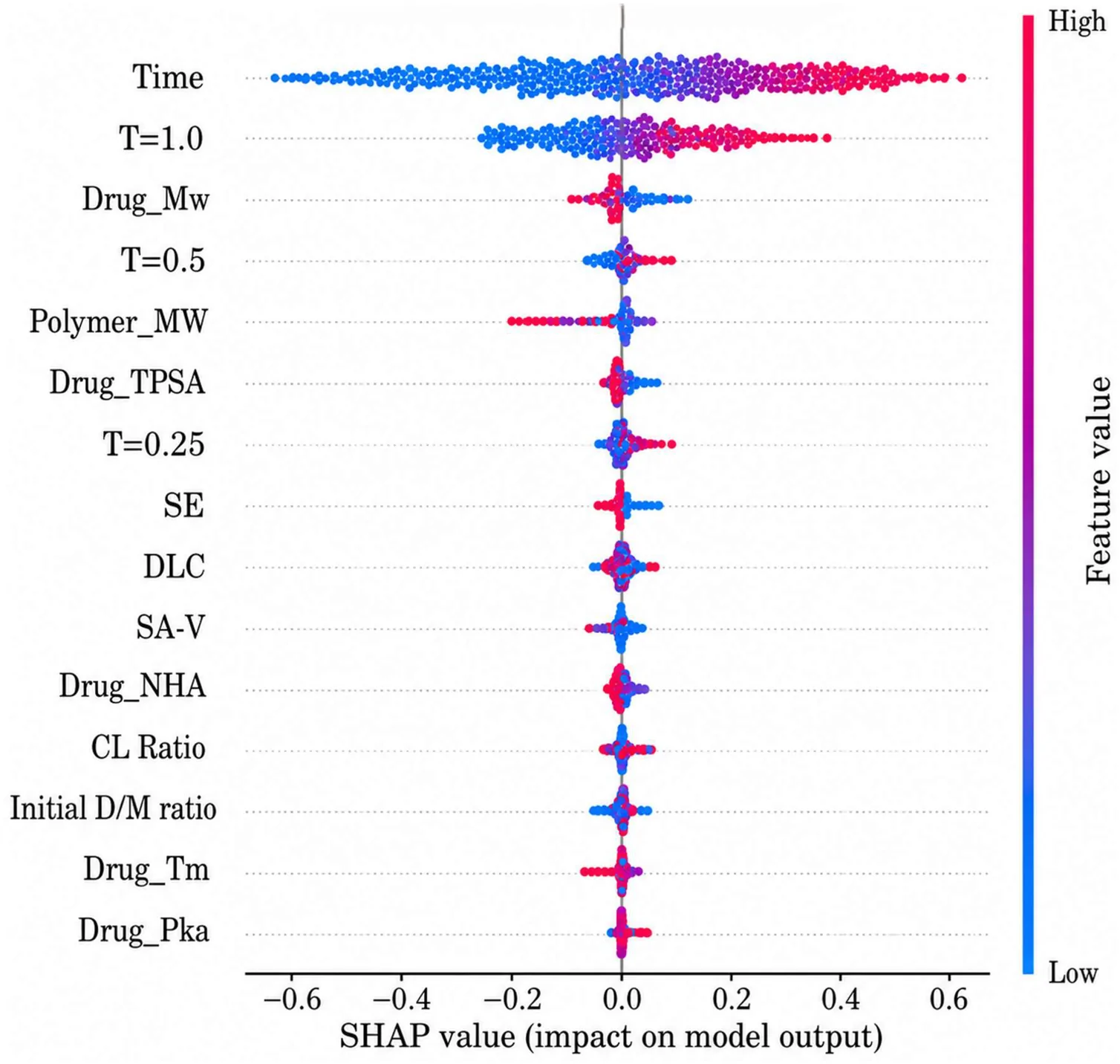
SHAP beeswarm plot of feature effects on predicted drug release.

### 4.5 Model-based virtual formulation analysis

Table 5 shows the effect of release time as its value changes over percentile values without altering the rest of the variables at their baseline levels. The percentile values (P0–P100) were derived from the observed distribution of release time in the dataset; therefore, the evaluated time points remain within the empirical feature range and primarily represent interpolation rather than extrapolation. Predicted release increased steadily from almost zero at the lowest percentile point upwards to 0.890 at the highest percentile, with the median condition yielding a baseline prediction of 0.442. Put differently, the model forecast larger release values progressively as time increased, in accordance with the cumulativity of the drug release feature.

**Table 5:** Counterfactual change in predicted drug release relative to baseline across time percentiles.

Time percentile	Predicted release	Change from baseline	Change (%)
P0	−0.001	−0.443	−100.3%
P10	0.035	−0.407	−92.0%
P25	0.212	−0.230	−52.0%
P50 (baseline)	0.442	0	0%
P75	0.712	+0.271	+61.3%
P90	0.849	+0.408	+92.3%
P100	0.890	+0.448	+101.5%

Because the intervention values are restricted to the observed range, these scenarios provide a more practically grounded assessment of the model response than interventions involving values outside the training distribution. Nevertheless, the predicted outcomes remain model-based estimates and should be experimentally validated before guiding formulation decisions. These outcomes should be seen as model-based intervention analyses within the conditional framework assumption, so they should not be interpreted as direct causal evidence or as experimentally observed release profiles. The counterfactual analysis therefore serves primarily as a computational screening tool to evaluate formulation-relevant scenarios within the data-supported feature space and to identify conditions that may warrant subsequent laboratory investigation.

### 4.6 Limitations and future work

Although the knowledge-guided DAG and intervention analyses provide a structured basis for evaluating hypothetical formulation changes, the estimated counterfactual effects remain computational predictions and cannot substitute for experimental evidence of causality. Future work should therefore experimentally validate selected counterfactual scenarios, particularly those showing favorable predicted release changes, to determine whether the predicted intervention effects are reproducible under laboratory conditions and to further establish the practical utility of the proposed framework for formulation development.

## 5 Conclusion

The study presents a mechanism-informed ML framework that the research team created for forecasting drug release from polymeric long-acting injectable systems. The framework was created by merging different techniques such as predictive modeling, pharmaceutical knowledge, knowledge-guided graph creation, explainable artificial intelligence, effect-estimation methods, and model-based virtual intervention analysis under a cohesive pipeline. Instead of considering predictive accuracy as the top criterion, the novel framework separates intervention-oriented effect estimates (under an assumed knowledge-guided graph) from model-based predictive importance and statistical association. This distinction is more useful for analyzing formulation-release interdependence and ranking variables for further research. Beyond prediction accuracy, the principal contributions are revealed. Correlation- and SHAP-based feature importance were found to be highly aligned (r = 0.761), while correlation-based importance vs. intervention-oriented effect estimates (r = 0.132) and SHAP importance vs. intervention-oriented estimates (r = −0.021) barely agreed. This finding implies that even the variables identified as most influential to prediction are unlikely to drive the most significant changes if formulation modifications were introduced, further illustrating that relying purely on conventional explainable ML is limiting for formulation decision-making.

Knowledge-guided graphs not only provide a detailed representation of pharmaceutical relationships but also enable intervention-oriented effect estimation and virtual formulation analysis. On average, the effects estimated by the different estimators ranged between 0.1860 and 0.3790. Moreover, the heterogeneous conditional effect distributions implied that formulation variables have a significant impact in differing formulation contexts. The results should not be seen as experimental proof of causality but rather as evidence for intervention-oriented studies, provided the assumptions in the knowledge-guided graph are satisfied. The accompanying model-based virtual intervention analysis further showed that computers can support hypothetical formulation modifications before moving to laboratory experiments and provide practical guidelines on which formulation strategies to explore first.

Overall, drug release modeling has moved beyond prediction to become more transparent, mechanism-informed, and, most importantly, intervention-oriented by introducing drug formulation optimization. By combining predictive modeling, pharmaceutical knowledge, model interpretability, and virtual intervention analysis, the method here differentiates between things that predict drug release and what can be done about formulation optimization. To name a few things that should be further verified after this work, future research should verify the estimated intervention effects by means of experimental trials, assess the framework with the help of independent formulation datasets, factor in uncertainty quantification, as well as develop stronger graph-learning techniques to strengthen the robustness and generalizability of the design of the intelligent drug delivery system.

## Data Availability

The original contributions presented in the study are included in the article/Supplementary Material, further inquiries can be directed to the corresponding author.
